# Increasing Heat Transfer from Metal Surfaces through Laser-Interference-Induced Microscopic Heat Sinks

**DOI:** 10.3390/mi14091730

**Published:** 2023-09-02

**Authors:** Frederic Schell, Richard Chukwudi Okafor, Tobias Steege, Sabri Alamri, Savan Ghevariya, Christoph Zwahr, Andrés F. Lasagni

**Affiliations:** 1 Fraunhofer Institute for Material and Beam Technology IWS, Winterbergstr. 28, 01277 Dresden, Germany; 2 Fusion Bionic GmbH, Löbtauer Str. 69, 01159 Dresden, Germany; 3 Faculty of Mechanical Engineering, Technische Universität Dresden, Georg-Bähr-Str. 3c, 01069 Dresden, Germany

**Keywords:** direct laser interference patterning, microstructures, heat transfer, heat sink, stainless steel, nanosecond

## Abstract

With the increasing processing power of micro-electronic components and increasing spatial limitations, ensuring sufficient heat dissipation has become a crucial task. This work presents a microscopic approach to increasing the surface area through periodic surface structures. Microstructures with a periodic distance of 8.5 µm are fabricated via Direct Laser Interference Patterning (DLIP) on stainless steel plates with a nanosecond-pulsed infrared laser and are characterized by their developed interfacial area ratio. The optimal structuring parameters for increasing the surface area were investigated, reaching peak-to-valley depths up to 12.8 µm and increasing surface area by up to 394%. Heat dissipation in a natural convection environment was estimated by measuring the output voltage of a Peltier element mounted between a hot plate and a textured sample. The resulting increase in output voltage compared to an unstructured sample was correlated to the structure depth and developed interfacial area ratio, finding a maximum increase of 51.4%. Moreover, it was shown that the output voltage correlated well with the structure depth and surface area.

## 1. Introduction

In recent decades, computing power has increased significantly due to decreasing transistor size; therefore, there are now more transistors per chip. For processing units to operate at their maximum capacity, one factor has especially gained relevance: the operating temperature. Controlling the temperature is widely regarded as the most crucial parameter to prevent microelectronic device failure and ensure reliable operation [1]. Electronic devices are, therefore, conventionally cooled using heat sinks built from materials with high thermal conductivity and designed to maximize surface area, e.g., by an array of fins. However, with small processing units and embedded systems becoming more common in recent years, the use of macroscopic heat sinks has become less feasible in many applications due to the limits of installation space. It is, therefore, of interest to increase heat transfer without macroscopic surface extension.

Heat transfer from surfaces works by two distinct phenomena: convection and radiation. By controlling the surface roughness of a material, the heat transfer from the surface can be adjusted by influencing surface properties that affect both modes of heat transfer [2]. Many studies have shown a beneficial effect of surface area on heat transfer [2,3,4,5]. Firstly, surface roughness is related to surface area. Since heat transfer by means of both convection and radiation is directly proportional to the surface area from which the heat is conducted, increasing the surface area can greatly increase the heat transfer of both transfer modes. Moreover, radiative heat flux depends on the emissivity of the surface, which itself is a function of the surface properties and, therefore, surface roughness. It follows that the macroscopic approach should also apply to the microscopic domain, as has been shown by multiple studies observing increased heat transfer after surface micro- and nanostructures were applied [6,7].

One technique that lends itself particularly well to the controlled and cost-effective generation of surface roughness is laser texturing. There already exists a considerable amount of studies demonstrating the effectiveness of laser surface textures in increasing pool boiling heat transfer [8,9,10,11,12]. However, in the case of boiling heat transfer, the increase is at least partially attributed to the influence of the surface textures on bubble nucleation [9]. Nonetheless, other studies on laser surface textures have observed increased heat transfer in a natural convection environment. For instance, Ventola et al. [13] investigated the heat transfer from laser-sintered heat sinks. They found a maximum increase of 73% due to the roughness of the sintered surface in comparison to flat surfaces. Using femtosecond lasers, Vorobyev et al. developed a back metal surface through laser-induced oxidation, which increased emission efficiency up to 100% [14]. Moreover, by applying a similar laser patterning, researchers were able to increase the output of a solar-driven thermoelectric generator (TEG) by increasing its absorptance in the ultraviolet and visible spectrum [15]. Similarly, the blackened metal increased the output power of a conventional TEG by 280% as a consequence of increased surface area and emissivity [16].

In the domain of laser texturing, Direct Laser Interference Patterning (DLIP) has recently been gaining popularity as an alternative to conventional laser direct writing due to the possibility of achieving significantly higher throughput with specialized optical configurations (Lang et al.: 0.9 m^2^/min [17], Ränke et al.: 1.1 m^2^/min [18]). It also offers the ability to circumvent feature resolution limits of conventional techniques by producing periodic structures inside the area of a single laser pulse. This result is achieved by overlapping multiple sub-beams on the substrate surface that form an area of interference in which a periodic distribution of intensity arises. The target material is thus only ablated around the interference maxima, where the material-dependent threshold fluence is exceeded. The geometry of the interference pattern depends on the number of interfering beams, their wavelength, their phase, and their polarization [19,20]. For two beams with equal intensity, phase polarization, and equal angle towards the optical axis, the spatial period Λ of the intensity pattern only depends on the chosen laser wavelength and the angle θ between the beams.

Due to the aforementioned unique characteristics, DLIP is well suited to mass-produce laser-textured heat exchangers. While the effectiveness of direct write techniques has already been demonstrated, there is a lack of studies investigating the influence of DLIP textures with dimensions in the low-micrometer range on heat transfer. We performed these experiments on stainless steel since good structure homogeneity is harder to obtain on copper and aluminum with an infrared nanosecond laser source.

Therefore, this work uses nanosecond DLIP to texture stainless steel surfaces with varying surface roughness and surface area. Using a Peltier element, the heat transfer of the textured samples is estimated by measuring the output voltage of the Peltier element mounted to a heating plate.

## 2. Materials and Methods

### 2.1. Materials

For the experiments, the austenitic chromium–nickel steel 1.4301 X5CrNi18-10 (SG Designbleche GmbH, Erkelenz, Germany) was selected due to the widespread use of steel in the industry and the higher quality of laser-generated structures on steel compared to other metals, such as copper and aluminum, under similar conditions [1]. The untextured surface had a roughness (Sa) of 10.0 ± 1.9 nm and developed interfacial area ratio (Sdr) of 0.4 ± 0.19%. Steel samples with an area of 40 × 40 mm^2^ and a thickness of 0.8 mm were cut out to investigate the heat transfer characteristics. All surfaces were rinsed with ethanol prior to the laser treatment and were textured on one side only. 

### 2.2. Laser Texturing

**Figure 1:**
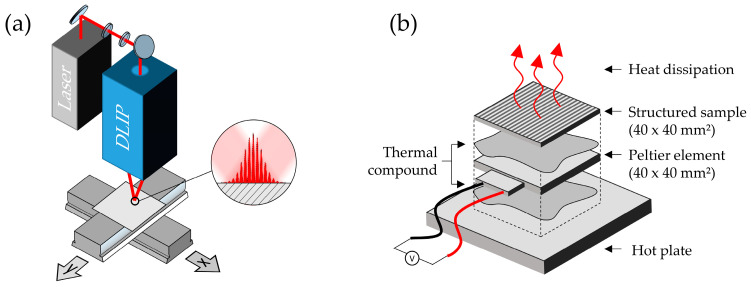
Schematic of (**a**) the texturing setup consisting of a nanosecond-pulsed laser, a DLIP optical head, and two translational stages as well as (**b**) the heat transfer setup consisting of a textured sample mounted on a Peltier element, which is in turn mounted on a hot plate with layers of thermal compound in between.

All DLIP-textured samples were produced using a two-beam interference setup powered by an IR (1053 nm) Q-switched diode-pumped solid-state laser (Laser Export, TECH-1053, Moscow, Russia) with a pulse duration of 15 ns, a repetition rate of 1 kHz, and a maximum pulse energy of 500 µJ. The system employed for the manufacturing process was a self-developed compact DLIP system (DLIP-µFab, Fraunhofer IWS, Dresden, Germany, shown schematically in Figure 1a). It consists of an optical head in which a a single laser beam is split by a diffractive optical element into two sub-beams that are superimposed on the substrate surface by an aspheric converging lens, thus creating an interference pattern in the overlapping volume of the beams. The interference diameter (d_i_) in the largest cross-section was estimated to be 121.2 µm using the D-squared method [21]. The angle between the beams was adjusted to 7.1°, forming an interference pattern with a spatial period (Λ) of 8.5 µm. Samples were translated between laser pulses through motorized linear stages (Aerotech PRO155-05, Pittsburgh, PA, USA). The axes controller triggered the laser externally to achieve a constant pulse distance (p) in the direction parallel to the interference fringes. The number of pulses per spot (N) along that direction was calculated as follows:

(1) $$N=p/d_{i}$$

Larger areas were textured by overlapping lines of pulses containing the interference structures, where the hatch distance (hd) defined the distance between adjacent lines of pulses and was set to an integer multiple of the spatial period. Pillar-like textures were achieved by subjecting a micro-channel-textured specimen to a second texturing process perpendicular to the first processing step. To obtain approximately equal structure depths in both axes, the fluence ($\Phi_{1}$) used in the first prossessing step was reduced to a empirically determined lower fluence ($\Phi_{2}$) for the second processing step.

### 2.3. Surface Characterization

The surface topography was measured utilizing confocal laser-scanning microscopy (CLSM, Keyence, VK-X3000, Osaka City, Japan), using a 50× magnification objective. Surface roughness and surface area were estimated using the software package MountainsMap (Digital Surf, Version 6.2.7487) according to the ISO 25178 standard [22]. High-resolution imaging of the surface textures was conducted using a scanning electron microscope (SEM) at 15 kV operating voltage (Jeol, JSM 6610LV, Akishima City, Japan). The surface oxygen content was estimated using energy dispersive X-ray spectroscopy (EDX) measurements using an EDX detector (Oxford Instruments, Ultim Max 100, Abingdon, UK) on a second SEM (Jeol, IT700). For each sample, an area of 0.9 × 1.2 mm^2^ was probed using a low accelerating voltage of 3kV to achieve a low interaction depth estimated to be around 100 nm. Quantitative analysis was performed using AzTec software (Oxford Instruments, Version 6.1) to obtain the oxygen mass fractions. In the case of channel structures, the channels were oriented parallel to the plane spanned by the detector axis, and the sample surface normal. 

### 2.4. Heat Transfer

Indirect heat transfer measurements were conducted using a Peltier element (Laird Thermal Systems, HiTemp ETX Series ETX5-16-F1-4040-TA-W8, Morrisville, NC, USA) with a 40 × 40 mm^2^ area. The Peltier element was placed on a temperature-controlled heating plate with its hot side, using a thermal compound (Arctic MX-2, Braunschweig, Deutschland) as a mediating layer, as shown in Figure 1b. Laser textured samples with the same lateral dimensions were mounted on the cold side of the Peltier element with their untextured side, again with an intermediate layer of thermal compound. After the temperature of the hot plate reached its setpoint of 100 °C, the voltage of the Peltier element generated by the Seebeck effect was measured using an analog-digital converter (USB-6009, National Instruments, Austin, TX, USA). After a steady-state heat transfer condition was reached (after waiting a minimum duration of 15 min), the voltage was recorded over a duration of at least 10 min at a sample rate of 20 Hz. The voltage value for each texture was calculated from the average voltage over the measurement interval.

## 3. Results and Discussion

### 3.1. Laser Texturing

**Figure 2:**
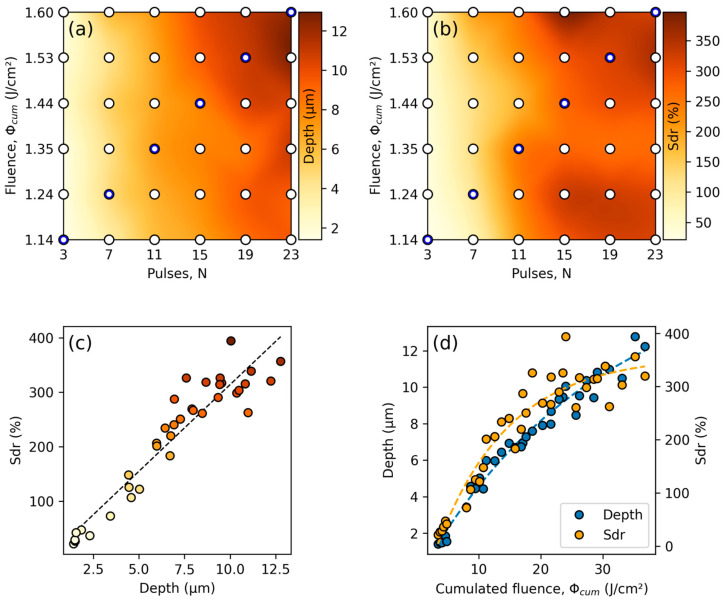
Plots of the micro-channel textures’ (**a**) average peak-to-valley depth as a function of the number of pulses and pulse fluence, (**b**) developed interfacial area ratio (Sdr) as a function of the number of pulses and pulse fluence, (**c**) correlation between developed interfacial area ratio and peak-to-valley depth (R = 0.94), and (**d**) depth and developed interfacial area ratio as a function of the cumulated fluence. The white dots in (**a**,**b**) indicate the measured values, whereas values in between are interpolated. The color in (**c**) encodes the values of Sdr.

Different micro-channel textures were fabricated on a stainless-steel sample using a spatial period of 8.5 µm. To fabricate channels with different peak-to-valley depths, the laser fluence and number of pulses per spot were varied systematically while fixing the hatch distance to 34 µm. The fluence varied between 1.14 and 1.60 J/cm^2^, and the number of pulses varied between 3 and 23 pulses, corresponding to a pulse overlap of 66.67% and 95.65%, respectively. A 1 × 1 mm^2^ texture was fabricated for each pair of parameters at a fixed laser repetition rate of 1 kHz. The textures were measured using a confocal microscope, and the average peak-to-valley depth was estimated from a series of 40 profiles perpendicular to the micro-channel orientation. As shown in Figure 2a, the depth increases from a minimum of 1.42 µm at the lowest number of pulses and fluence to a maximum of 12.78 µm at the highest employed number of pulses and fluence. The gradient is notably smooth, which indicates that the parameter window is not large enough to detect a global maximum. As a corresponding measurement of surface area, the developed interfacial area ratio (Sdr), defined by ISO 25178, is computed for all data points and shown in Figure 2b. Sdr represents the increase in surface area compared to the projected area (area of an indeally flat sample with same lateral dimensions). A maximum surface area increase of up to 394.1% is observed for N = 15 and Φ = 1.60 J/cm^2^. Values of Sdr follow a similar trend as the structure depth, generally increasing for an increasing number of pulses and pulse fluence. However, some fluctuations are visible, and Sdr values follow a larger gradient as a function of the number of pulses than the pulse fluence. The fluctuations can be explained by the strong dependence of Sdr on the nanoroughness, debris, and melting artifacts. Nonetheless, Sdr correlates to a high degree (R = 0.94) with the structure depth in the chosen parameter window, as elucidated in Figure 2c. Plotting the peak-to-valley depth and Sdr against the cumulated fluence (Figure 2d) obtained by multiplying the number of pulses with the pulse fluence, it can be observed that, in the chosen parameter window, the cumulated fluence is the main factor determining the structure depth and surface area. The structure depth exhibits a nearly linear increase, whereas the developed interfacial area ratio already shows a saturation behavior in the cumulated fluence regime above 20 J/cm^2^. 

Samples along the diagonal of the texture matrix (from Φ = 1.14 J/cm^2^, N = 3 to Φ = 1.60 J/cm^2^, N = 23), which represent a nearly constant increase in cumulated fluence and consequently in depth, were selected for heat transfer measurements. With each of these six parameter pairs, a 40 × 40 mm^2^ sample was textured using the parameters given in Table 1. Moreover, a second set of samples with micropillar textures was fabricated using a second irradiation step perpendicular to the first processing step. For this purpose, the fluence in the second processing step (Fluence B in Table 1) was adjusted to a value resulting in isotropic depth profiles in both axes. 

**Figure 3:**
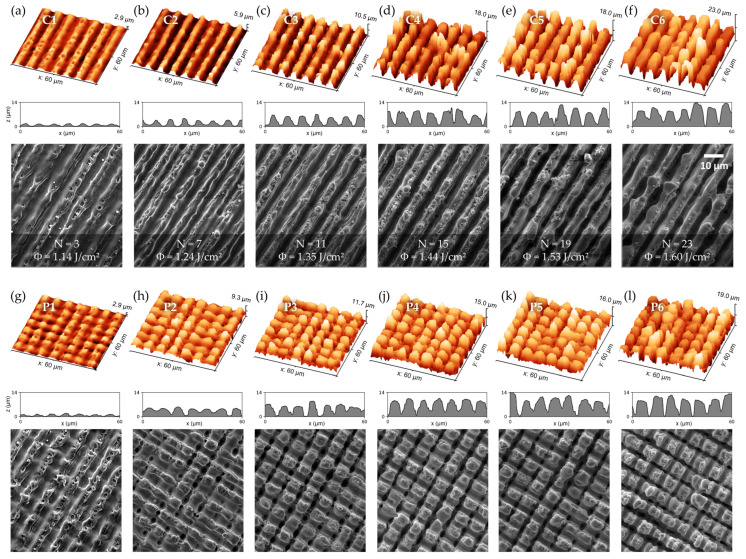
CLSM measured 3D-topographies, structure profiles, and SEM images of selected microtextures fabricated with increasing cumulated fluence from left to right. The upper sequence (**a**–**f**) depicts micro-channel structures fabricated by a single processing step. The lower sequence (**g**–**l**) depicts micro-pillar structures fabricated by a second irradiation process oriented perpendicular to the first one.

These samples were imaged using a CLSM and an SEM. The results are shown in Figure 3a–f for micro-channel and Figure 3g–l for micro-pillar structures. Due to the nanosecond-pulse interaction with the material, the formation process of the structures is characterized by substantial melting. For a low fluence and number of pulses, the recast material from the regions of two adjacent interference maxima positions solidifies on the regions of the interference minima. However, it does not fully merge, leaving melt protrusions and not fully bonded structure peaks (Figure 3a,g). A full bonding of the peaks occurs when increasing the fluence and number of pulses (Figure 3b–d,h–k). For the highest fluence and number of pulses (Figure 3e,f,k,l), the large amount of expulsed molten phase leads to the building of slightly overhanging spherical structure peaks in some locations and even full bridging of adjacent structure peaks. 

**Figure 4:**
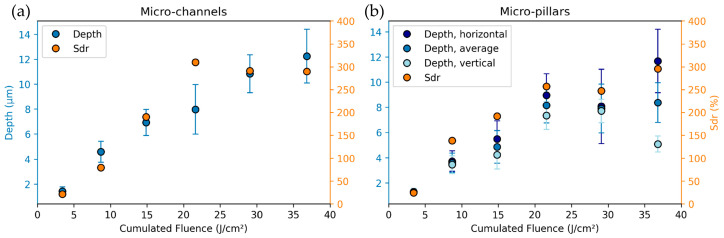
Depth and Sdr of (**a**) micro-channel and (**b**) micro-pillar type textures as a function of the cumulated fluence.

The structure depth and Sdr of the samples fabricated for heat transfer measurements are given in Figure 4a for micro-channel structures and Figure 4b for micro-pillar structures. Similar to the results shown in Figure 2, a correlation between depth and Sdr is observed, as well as a saturation behavior of Sdr for cumulated fluences above 20 J/cm^2^. In the case of the micro-pillars, the depth is evaluated in both texture axes (horizontal and vertical), whereby the horizontal axis corresponds to the first processing step and yields similar values as the micro-channel structures. The depth in the vertical axis was significantly lower than in the horizontal axis for micro-pillars fabricated with cumulated fluences above 20 J/cm^2^, indicating that the second processing step’s chosen process parameters are not sufficient enough to result in isotropic depth profiles.

**Figure 5:**
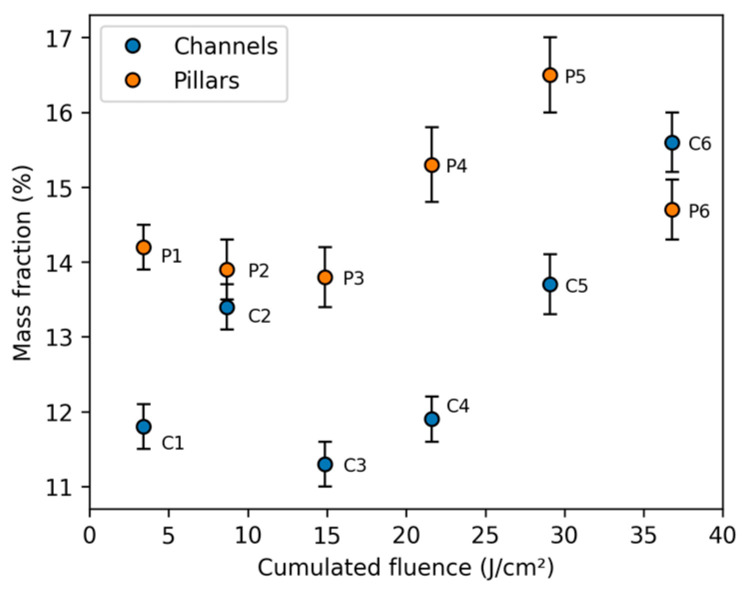
Oxygen atomic fraction of channel (C1–C6) and pillar textures (P1–P6) obtained by EDX analysis. The interaction depth was approximately 100 nm, and the detector axis was oriented along the channels.

To estimate the surface oxidation as a result of the DLIP texturing process, EDX analysis is conducted on the six channel and six pillar textures as well as on the untextured reference, and the oxygen mass fraction is computed and given in Figure 5. Since the absolute values obtained from the EDX technique are only valid for flat homogenous surfaces, the mass fractions given in Figure 5 can only be used as a basis for qualitative analysis. The untextured reference exhibits a close to 0% oxygen mass fraction. However, due to the overlapping chromium and oxygen peaks, low oxygen mass fractions cannot be reliably detected in the presence of a significant amount of chromium in the steel. For the textured samples, it is observed that the oxygen content on both the channel and pillar textures remains relatively constant up until a cumulated fluence of 15 J/cm^2^ (C3, P3), except for C2, which could be related to a local inhomogeneity and, therefore, be classified as an outlier. 

For higher cumulated fluences (C4–C6, P4–P6), there is a noticeable increase in the surface oxygen content. Overall, the pillar textures exhibit significantly higher oxygen content than their channel counterparts, which is most likely related to the additional exposure to the laser radiation in the second processing step (which is not reflected in the cumulated fluence of the graph). The oxygen content is only weakly correlated to the cumulated fluence (channels: 0.71, pillars: 0.61), structure depth (channels: 0.66, pillars: 0.60), and developed surface area (channels: 0.44, pillars: 0.60). However, it cannot be excluded that the low degree of correlation could be a consequence of the flaws of EDX measurements on non-flat non-homogenous surfaces. Nonetheless, the higher surface oxidation on samples fabricated with larger cumulated fluences (C4–C6, P4–P6) is likely to increase the emissivity of the surfaces and could thereby increase heat transfer through radiative cooling [23]. 

### 3.2. Heat Transfer

**Figure 6:**
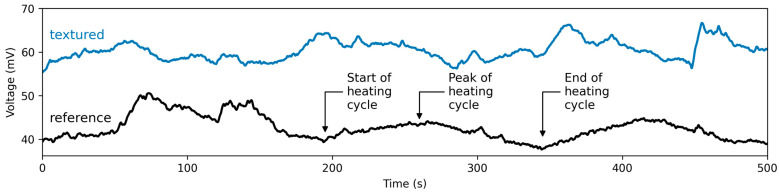
Exemplary voltage signal of an untextured reference (black) and textured (Texture C5) specimen (blue) recorded over a duration 500 s. The high-frequency signal components were removed using a Butterworth low-pass filter for visual purposes.

The heat dissipation from the micro-channel and micro-pillar structures was estimated by measuring the output voltage of a Peltier element placed between a textured sample and a hot plate. The temperature of the hot plate was set to 100 °C. After reaching a presumed steady-state heat transfer condition, the voltage was recorded. Since, according to the Seebeck effect, the voltage is approximately proportional to the temperature difference between the element’s hot and cold sides, it can be used to qualitatively estimate the heat conduction through the element, which is also proportional to the temperature difference. Figure 6 shows two selected voltage signals recorded over a duration of 500 s for the untextured reference and a micro-channel textured sample. The voltage fluctuates over the measurement interval primarily due to the cyclic heating caused by the control loop of the hotplate, as indicated for the untextured reference sample in Figure 6. The average voltage of the untextured reference was 42.3 ± 2.7 mV, and all textured samples surpassed the output of the untextured reference. 

**Figure 7:**
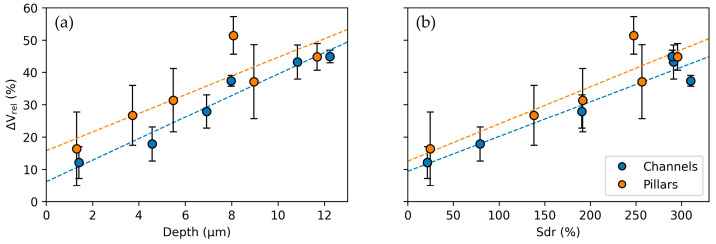
Relative increase of the output of the Peltier element for textured surfaces compared to an untextured surface as a function of (**a**) the structure depth (horizontal axis) and (**b**) the developed interfacial area ratio (Sdr). The heating plate is set to a fixed temperature of 100 °C.

The Peltier element’s output voltage increase relative to the reference value is given in Figure 7 as a function of the structure depth (Figure 7a) and the developed interfacial area ratio (Figure 7b) for both micro-channels and micro-pillars. For both types of textures, the output voltage increases when the structure depth of Sdr is increased. Micro-channel textures reached a maximum increase of 44.9% for a depth of 12.2 µm and Sdr of 320.2%, whereas micro-pillar textures exhibited a maximum increase of up to 51.4% for a depth of 10.8 µm and an Sdr of 315%. Moreover, the output voltage follows a strong linear trend as a function of structure depth and Sdr for micro-channel (R_Depth_ = 0.98, R_Sdr_ = 0.97) and micro-pillar textures (R_Depth_ = 0.86, R_Sdr_ = 0.90), exhibiting high coefficients of correlation. A stronger correlation is observed as a function of the structure depth. This result can be explained by the more deterministic nature of the depth as opposed to Sdr, as the latter is also strongly influenced by nanoroughness, melt protrusions, and other deviations from the ideal surface pattern. Notably, there is no substantial difference in the heat transfer between microchannel and micropillar structures. Micropillar structures slightly outperform the microchannel structures, but the difference is not significant. This outcome could be related to the hindrance of heat dissipation from the deep valley regions of the pillar structures by more pronounced overhanging elements on the peaks, as observed in Figure 3. Moreover, the scattering of the values could be related to unpredictable air turbulences above the sample surface due to the heat source being placed beneath the samples.

From these results, it remains unclear how much of the increased heat dissipation from the structures is a consequence of the increased surface area and thus related to the natural convection heat transfer mode and how much is associated with the increased surface emissivity. Even though the surface area (quantified by Sdr) correlates well with the increase in output voltage and, thus, heat dissipation, the oxidation level of the surface also correlates with the cumulated fluence, which in turn correlates with the increased surface area. Therefore, increased oxidation and, thus, darker surface discoloration due to the laser process could increase emissivity significantly and enhance the heat dissipation by the radiative transfer mode.

## 4. Conclusions

In this work, nanosecond two-beam Direct Laser Interference Patterning (DLIP) was used to fabricate micro-channels and micro-pillars with a spatial period of 8.5 µm and varying peak-to-valley depth as well as surface area. By varying the pulse fluence and number of pulses, peak-to-valley depths up to 12.78 µm and increased surface area by up to 394% could be obtained. 

It was shown that the surface area quantified by Sdr correlated to a high degree with the peak-to-valley depth and that the cumulated fluence was the determining parameter for the depth and surface area of the microstructures. The EDX measurements showed that high cumulated fluences resulted in higher surface oxidation during the laser process, possibly increasing surface emissivity. The heat dissipation performance in a natural convection environment was estimated by measuring the output voltage of a Peltier element mounted between a textured sample and a hot plate. It was observed that the voltage increased with increasing surface area and structure depth. A maximum increase of 51.4% was observed compared to the untextured reference sample. Moreover, the output voltage could be linearly tuned as a function of the structure depth or Sdr. These results demonstrate that the DLIP technique is well suited to fabricate microscopic heat sinks that could be applied in confined spaces for microelectronics, where macroscopic heat sinks would be impractical. The influence of substrate temperature and surface color will be evaluated in future investigations (e.g., an evaluation by oxidizing the steel surface with heat treatment). Also, ultrashort pulse green lasers will be used to fabricate similar structures on copper.

## Figures and Tables

**Table 1:** Laser process parameters for the fabrication of micro-channel and -pillar textures. Microchannel textures are fabricated with fluence A. Pillar textures are fabricated using a second processing step by changing the fluence from fluence A to fluence B but keeping the number of pulses and hatch distance.

Texture	Label	Fluence A (J/cm^2^)	Fluence B (J/cm^2^)	Pulses	Hatch Distance (µm)
Channels	C1	1.14	-	3	34
Channels	C2	1.24	-	7	34
Channels	C3	1.35	-	11	34
Channels	C4	1.44	-	15	34
Channels	C5	1.53	-	19	34
Channels	C6	1.60	-	23	34
Pillars	P1	1.14	1.04	3	34
Pillars	P2	1.24	1.07	7	34
Pillars	P3	1.35	1.17	11	34
Pillars	P4	1.44	1.21	15	34
Pillars	P5	1.53	1.28	19	34
Pillars	P6	1.60	1.04	23	34

## Data Availability

Data are available upon reasonable request to the authors.
